# Tailored interventions for inappropriate psychotropic drug use in nursing home residents with dementia: participatory action research in a special case of a stepped-wedge cluster randomized controlled trial

**DOI:** 10.1186/s12877-025-06206-y

**Published:** 2025-08-02

**Authors:** Claudia M. Groot Kormelinck, Debby L. Gerritsen, Charlotte F. van Teunenbroek, Michiel R. de Boer, Martin Smalbrugge, Sytse U. Zuidema

**Affiliations:** 1 Department of Primary and Long-term Care, University of Groningen, University Medical Centre Groningen, PO Box 196, Groningen, 9700 AD, FA21 the Netherlands; 2 Alzheimer Centre Groningen, University Medical Centre Groningen, Groningen, the Netherlands; 3 Department of Primary and Community Care, Radboud UMC Alzheimer Centre, Research Institute for Medical Innovation, Radboud University Medical Centre, Nijmegen, the Netherlands; 4 Department of Medicine for Older People, Amsterdam UMC, location Vrije Universiteit Amsterdam, de Boelelaan 1117, Amsterdam, the Netherlands; 5 Amsterdam Public Health Research Institute, Aging & Later Life, Amsterdam, the Netherlands

**Keywords:** Dementia, Nursing homes, Psychotropic drugs, Neuropsychiatric symptoms, Complex interventions

## Abstract

**Background:**

Psychotropic drugs are modestly effective and may cause adverse effects. Efforts to reduce inappropriateness and increase usage of psychosocial interventions often suffer from suboptimal implementation. The purpose of this study was to evaluate effectiveness of an innovative study using implementation promoting elements in nursing home residents with dementia and neuropsychiatric symptoms.

**Methods:**

A multicenter cluster randomized controlled trial with a special case of a stepped-wedge design with two arms and one stap was designed. The intervention comprised participatory action research, tailored information provision and external coaching, leading to the implementation of tailored action and implementation plans. The primary outcome was inappropriateness of psychotropic drug use (Appropriate Psychotropic Drug Use in Dementia [APID] index) and the secondary outcome was percentage of psychotropic drug use at baseline, 8 months, and 16 months. Homes were allocated to start with usual care or the intervention. After 8 months, the control group crossed over to receive the intervention. The other homes continued the intervention to 16 months. Patients were eligible if they were diagnosed with dementia, had a life expectancy of at least 3 months, and resided in psychogeriatric units.

**Results:**

An adjusted multilevel model revealed no effect on the APID index sum score at 8 months (0.564; 95% confidence interval [CI], -2.449–3.577; *p* = 0.71) or 16 months (2.165; 95% CI, -1.113–5.443; *p* = 0.20). An adjusted generalized estimation equation (GEE) model showed an effect at 16 months for percentage of use (OR 0.654; 95% CI, 0.481–0.889; *p* = 0.007). Adjusted GEE models showed an effect especially at 16 months for anxiolytics (OR 0.573; 95% CI, 0.382–0.859; *p* = 0.007) and antidepressants (OR 0.678; 95% CI, 0.475–0.968; *p* = 0.033).

**Conclusions:**

No reduction of inappropriateness was found although overall usage was reduced. Professionals focused on implementing alternatives to compensate for usage, rather than prescribing quality. Future studies may focus on changing physicians’ prescribing behaviors in combination with multicomponent and multidisciplinary psychosocial alternatives.

**Trial registration:**

Netherlands Trial Registry (NTR5872) on 27/05/2016, https://onderzoekmetmensen.nl/nl/node/26060/pdf.

**Supplementary Information:**

The online version contains supplementary material available at 10.1186/s12877-025-06206-y.

## Introduction

Dementia afflicts over 55 million people worldwide, with projections suggesting the potential for nearly 10 million new cases each year [1]. At some point, most people living with dementia will exhibit neuropsychiatric symptoms, such as depression, psychosis, agitation, aggression, apathy, and disinhibition. Estimates indicate that about 80% of nursing home residents in the Netherlands will experience at least one [2, 3]. Given that psychotropic drugs have only modest effectiveness at best and significant potential to cause side effects and adverse events [4–7], guidelines recommend psychosocial interventions as the first-line treatment [8–11]. Nevertheless, psychotropic drug usage remains prevalent [12, 13]. Regular use of at least one psychotropic drug is about 61%, whilst pro re nata use of psychotropic drugs is also common [14, 15]. In Western Europe nursing homes, for example, antipsychotics (range, 12–59%) and antidepressants (range, 19–68%) are being regularly prescribed [16]. Over the years, antipsychotic usage may have decreased somewhat, whilst benzodiazepine use may have increased. Moreover, the prescribing of psychotropic drugs may be considered inappropriate, for example regarding its indication, evaluation, and duration [14, 17–20].

Guideline recommendations [8] have led researchers to focus on reducing (inappropriate) psychotropic drug use and to increase the use of psychosocial and multidisciplinary multicomponent interventions for nursing home residents with dementia and neuropsychiatric symptoms [21–29]. This has produced mixed results, with some finding modest reductions in (inappropriate) psychotropic drug use [22–24, 26, 29] and others finding no change [25, 27]. When reductions occurred, the interventions generally produced relatively small effects [21, 22, 24, 25, 27]. Process evaluations have since uncovered barriers to suboptimal implementation, including high workloads, staff turnover, and lack of time to implement complex multicomponent interventions [30–34]. By contrast, engaging leaders, supporting key workers, and having a shared focus on change (i.e., specifically acceptance, commitment, and a positive attitude) may facilitate implementation [30, 31, 33]. However, especially the latter remains challenging. Agitation and aggressive behavior of residents may cause severe distress amongst nursing staff [35]. Physicians can feel pressured by nursing staff to prescribe psychotropic drugs as nursing staff may believe that the possible benefits outweigh any potential side effects [36] or there is a lack of trust in psychosocial interventions [37]. Discontinuation of psychotropic drugs may be impeded by fear of nursing staff for negative consequences [19]. Hence, it can be stated that creating a change can be challenging given the complex nature of nursing homes and concerning the potential attitudes and emotions that may play a role regarding this topic. As a result, ‘’one size fits all’’ standardized interventions are less likely to succeed and it is acknowledged that intervention and implementation should be tailored to emphasize the specific organizational contexts and addressing the culture, nature and characteristics of each organization [38–40]. The effectiveness of complex interventions within nursing homes may be improved by adapting interventions to local contextual barriers and facilitators [41, 42]. Employing a collaborative approach that engages multidisciplinary healthcare teams [33, 43] and provides guidance with opportunities for ongoing discussion and problem solving [43] may offer a solution. The Reducing Inappropriate psychotropic Drug use (RID) intervention was designed against this background. We hypothesized that interventions to reduce (inappropriate) psychotropic drug prescribing in nursing homes would benefit from a bottom-up approach with active involvement of staff in determining the problems and potential solutions, before tailoring the solution to the local setting with the support of an external coach. Participatory action research (PAR) can deliver precisely this type of collaborative and reflective strategy. It requires that researchers and participants work together to improve local practices by exploring and implementing potential solutions and making adjustments based on evaluations of their effectiveness in practice. Integrating this approach within a randomized controlled trial (RCT), known as PAR-RCT, can ensure generalizability [44]. Using this design, we evaluate whether tailored information provision and external coaching can produce action and implementation plans that reduce both inappropriate psychotropic drug use and the frequency of psychotropic drug use in nursing home residents with dementia. We also evaluate whether repeating the intervention cycle improves outcomes.

## Methods

### Study design

This multicenter cluster RCT with a special case of a stepped-wedge design with two arms and one step used a PAR approach in Dutch nursing homes and is part of the RID study. The full study protocol has been published elsewhere [45]. This report follows the CONSORT guidelines [46].

**Fig. 1:**
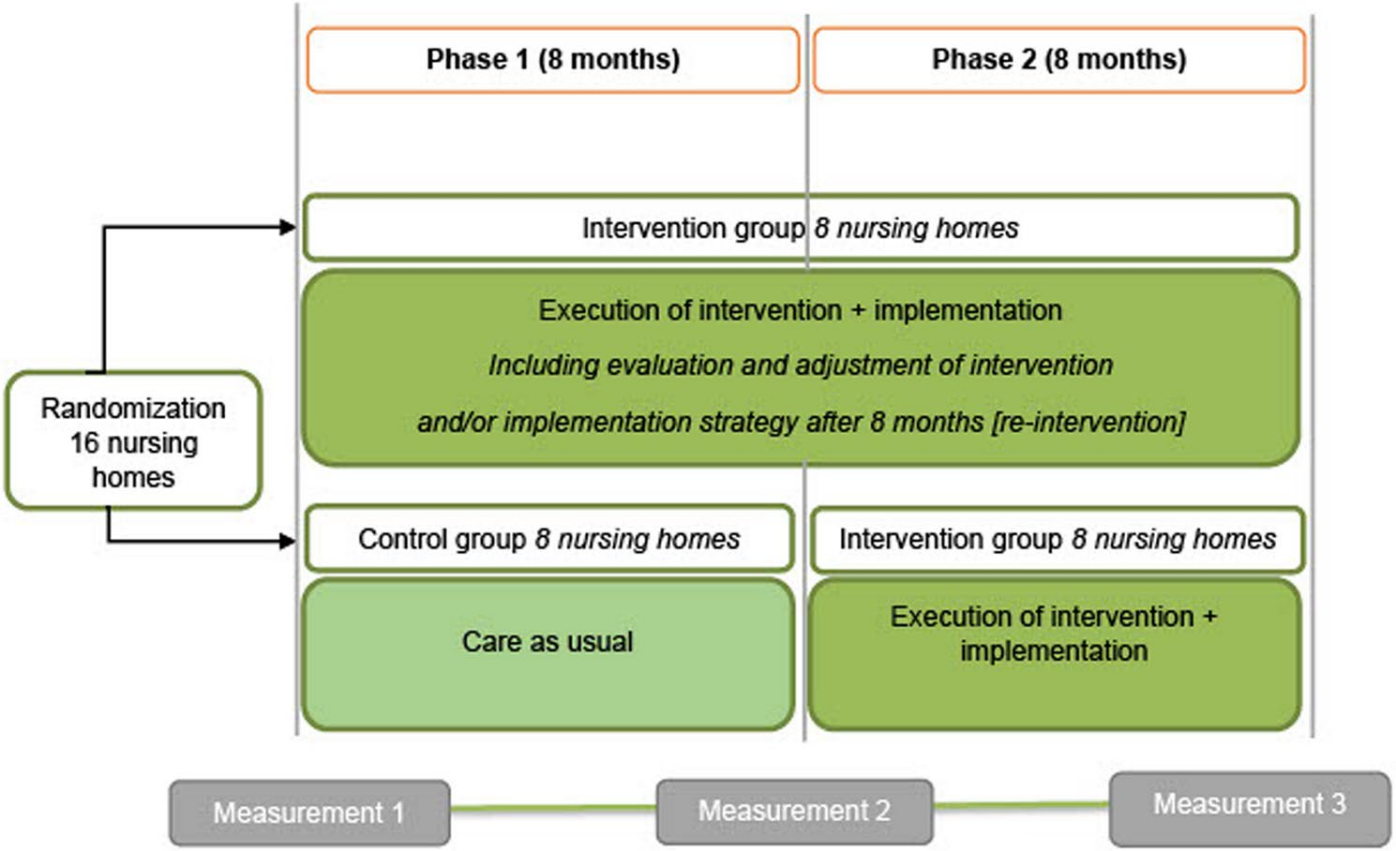
The RID Study: A special case of a stepped-wedge design with one step, two phases and three measurements RID = reducing inappropriate psychotropic drug use

The stepped-wedge design [47] had an overall duration of 16 months and comprised two 8-month phases, with measurements taken at baseline, 8 months, and 16 months. Phase one started with 16 nursing homes randomized to either the RID intervention group or the control group (usual care). Phase two started after 8 months with the nursing homes in the control group crossing over to the RID intervention group and the other eight nursing homes continuing with the RID intervention (Fig. 1). An independent statistician performed computer-generated blinded randomization in fixed blocks: round 1 (6 homes; blocks, 2-2-2) and round 2 (10 homes; blocks, 4-2-4) [45].

### Setting and participants

In the Netherlands, nursing homes provide dementia care in special care units (DSCUs). An elderly care physician typically has responsibility for any medical treatment, working in close collaboration with a psychologist, nurse practitioner, and nursing staff with varying levels of education and responsibilities. Homes may also employ physical, occupational, and activity therapists to improve wellbeing, functioning, and quality of life [48, 49]. In 2015, The Dutch government implemented a major reform aiming for elderly persons to stay as long as possible in their own homes. Residential care homes, taking care of elderly persons with moderate levels of impairment, were shutting down. Consequently, the threshold for admission to a nursing home increased. Only persons with complex health care problems in need of 24-hour surveillance and multidisciplinary care are eligible for admission. As a result, residents often have a quite short length of stay and relatively high mortality rates. In this respect, Dutch nursing homes may be different as compared to nursing homes in other countries [48].

We recruited nursing homes online after attending a national kick-off conference with presentations and an information market. An intake telephone call was then scheduled to assess the suitability of each home for inclusion, with 16 homes included by their order of application. DSCUs delivering care for residents with Korsakoff syndrome, acquired brain injury and Down’s syndrome were excluded. Units delivering care for young-onset dementia were also excluded. No age restrictions were imposed within the DSCUs providing care for residents with dementia at an older age. Each nursing home participated with a few large-scale units or multiple small-scale units. Nursing home residents were eligible for participation if they had a diagnosis of dementia and a life expectancy of at least 3 months, as judged by a physician. All eligible residents were approached for participation, including newly admitted residents, after the study began. More information can be found in the study protocol [45].

### RID intervention

A detailed description of the RID intervention can be found elsewhere [50]. The RID intervention involved forming a multidisciplinary project team with an internal project leader, a physician, a psychologist, and a nursing staff representative, together with a certified external coach to guide the cyclical process across four phases. Each intervention started with researchers executing a problem analysis on the management of neuropsychiatric symptoms and the appropriateness and percentage of current psychotropic drug use in their home (observation phase). The team then evaluated this tailored information and formulated specific goals under the guidance of the external coach (reflection phase), before operationalizing the goals into an action and implementation plan (planning phase). Finally, each nursing home implemented a set of interventions (action phase).

In some cases, there were differences between participating DSCUs within a nursing home, regarding the problem analysis or the potential solutions. Implementation was allowed to be tailored to a given DSCU, although in practice, most nursing homes developed and executed one action and implementation plan for all the participating DSCUs within their nursing home. The actions implemented by each nursing home varied based on their tailored problem analysis, but they generally targeted multidisciplinary and methodical working (including person-centered interventions), education and training, and adaptations to the living environment [50]. For the nursing homes that started in the RID intervention group in phase one, the measurement at 8 months was treated as an interim analysis that triggered the repetition of all four phases of the PAR cycle during the second phase of the trial (Fig. 1). Nursing homes that started in the control group in phase one provided care as usual for the first 8 months and entered an intervention cycle in phase two.

### Sample size

The sample size was based on the primary outcome (inappropriateness of psychotropic drug use). To detect a reduction of 5 points (standard deviation 15) on the Appropriateness of Psychotropic Drug Use in Dementia (APID) index with a power of 0.80, a two-sided α value of 0.05, and an average of 25 residents per nursing home, we estimated the need for 16 clusters (nursing homes). Not taking clustering into account, we needed to include 284 residents who used psychotropic drugs. However, allowing for the multilevel design with two measurements after baseline, an intraclass correlation coefficient of 0.1, a calculated design factor of 1.28, and a 10% cluster dropout, this increased to 364 residents. Given that an estimated 60% of residents with dementia are prescribed psychotropic drugs [17], we needed to include 607 residents (i.e., psychotropic drug users and non-users). We attempted to mitigate the expected 40% loss to follow-up by enrolling newly admitted residents throughout the study [45].

### Outcomes and data collection

Data on age, sex, dementia diagnosis, length of stay in the current DSCU, and number of psychotropic drugs were collected from each participant’s medical record. Both outcomes (inappropriateness– and percentage of psychotropic drug use) were also extracted from the medical records of residents. A team of (junior) researchers with educational backgrounds in medicine, psychology and health sciences collected data. The research team together pilot tested scoring of inappropriate psychotropic drug use by means of the APID index. Psychotropic drug usage included prescriptions of antipsychotics, anxiolytics, hypnotics, antidepressants, anticonvulsants and anti-dementia drugs. Anticonvulsants and antidementia drugs are listed as psychotropics drugs because they could have been prescribed to treat agitation in dementia and psychosis in Lewy Body dementia, respectively. Psychotropic drugs were grouped according to the Anatomical Therapeutic Chemical classification [51]. We excluded psychotropic drugs used pro re nata. If residents died or relocated more than 2 months after the measurements at baseline or 8 months, we collected any recorded data on psychotropic drug use at the next measurement.

The primary outcome was the inappropriateness of psychotropic drug use, as measured with the APID index. The APID index was developed by an expert panel based on the items of the Medication Appropriateness Index. The index has been evaluated among DSCU residents in the Netherlands [52, 53]. The APID rates the appropriateness of psychotropic drug use for residents with neuropsychiatric symptoms and dementia. Therefore, psychotropic drugs given for dementia, sleeping disorders, or delirium are included in the scoring, but those given for other psychiatric disorders are excluded. The APID instrument contains seven domains: indication, evaluation, dosage, drug-drug interaction, drug-disease interaction, duplication, and therapy duration. Using data from medical records, each domain is scored 0, 1, or 2 to reflect “appropriate,” “marginally appropriate,” and “inappropriate” usage, respectively. During the development, an expert panel weighted the relative importance of each single domain on a scale from one to ten, resulting in different ranges per domain: indication (range 0-18.8), evaluation (range 0-19.2), dosage (range 0-13.4), drug-drug interactions (range 0-11.6), drug-disease interactions (range 0-13.2), duplication (range 0-14.4), and therapy duration (range 0-12.2). These single domains can be incorporated into a weighted sum score using mean weights. The APID sum score ranges from 0 (fully appropriate) to 102.8 (fully inappropriate) per rated psychotropic drug. Hence, lower scores indicate more appropriate psychotropic drug use [52]. The APID index applies different rules regarding the indication and evaluation domains for prescriptions that are started prior to nursing home admission and for prescriptions started at the DSCU of the nursing home. For example, for psychotropic drugs that are started at the current DSCU the normal rules apply: a (correct) indication needs to be found within two months after starting the psychotropic drug. To assess the indication of a psychotropic drug that is started before admission to the DSCU, a 6-month period is allowed. Moreover, the indication is still considered appropriate even if an indication is lacking or incorrect if the 6-month period has not yet expired. The rationale behind this, according to the expert panel that developed the APID index, was that the physician should be given enough time to set an indication and to evaluate the usage of psychotropic drugs that were prescribed prior to nursing home admission.

The secondary outcome was the percentage of psychotropic drug use, evaluated as a binary variable (i.e., yes/no).

Data about neuropsychiatric symptoms were collected using the Neuropsychiatric Inventory-Nursing Home version (NPI-NH) [54]. A member of the nursing staff filled in paper versions of the questionnaire in the presence of a researcher. The NPI-NH assesses the frequency (score, 1–4), severity (score, 1–3), and caregiver distress (score, 0–5) for 12 psychiatric and behavioral symptoms. Item scores are generated by multiplying the frequency and severity [1–12], with possible scores ranging from 0 to 144, where a higher score indicates more frequent and severe neuropsychiatric symptoms [55].

### Statistical analysis

IBM SPSS, version 25 (IBM Corp., Armonk, NY, USA), was used to prepare the datasets and perform the descriptive statistics. Stata software, version 17.0, was used for all other analyses. Descriptive statistics were used to summarize the characteristics of residents at baseline by treatment arm, with data included for newly recruited residents at 8- and 16-months’ follow-up.

For the primary outcome, data was used from the residents using psychotropic drugs, with single psychotropic drug prescriptions as the level of observation. We compared the inappropriateness of psychotropic drug use between the intervention and control groups using multilevel models to accommodate the hierarchical data structure. These models were used to adjust for the clustering of residents within nursing homes (random intercept at the nursing home level) and for the correlation of the repeated measures and multiple prescriptions within residents (random intercept at the resident level). The dependent variable was set as the change in APID index score between two consecutive measurements. The analysis was adjusted for the number of psychotropic drugs per resident, sex, baseline NPI-NH total score, length of stay in the DSCU at baseline (in months), and time in the study arm. Residents were evaluated in four groups: full duration, later enrollment, early drop out, and later enrollment with early drop out. Time and the interaction of time with treatment were included as fixed effects. The model compared changes in the APID index sum score between baseline and either 8- or 16 months. Multilevel models were fitted with the restricted maximum likelihood method, and effect estimates are presented with 95% confidence intervals (CIs) and p values. Newly admitted residents were included at 8- and 16-month’s follow-up, but, considering that change scores were used for the primary outcome, data was only taken into account when residents were included in at least two measurements.

A different dataset and structure were used to evaluate the secondary outcome, percentage of psychotropic drug use. This dataset included all residents (psychotropic drug users and non-users) with observations at the resident level. Data of residents included at 8- and 16-month’s follow-up was taken into account. Psychotropic drug use between the control and intervention groups was compared by logistic generalized estimating equations (GEE), accounting for the clustering of repeated measurements within residents. GEE was used because it generates population average estimates that are preferable for intervention studies [56]. The model contained psychotropic drug use (yes/no) at 8 and 16 months as the dependent variables and assessed the main effect by group (intervention vs. control). We intended to correct for baseline NPI-NH sum score and baseline psychotropic drug use. Given the possibility of collinearity between these variables, they were added to the model one by one. Many residents were not included at the baseline measurement, which led to missing data; however, imputation was not feasible because the data concerned the period before admission. Two GEE models were ultimately executed: (1) analysis of all cases without correction for the NPI-NH sum score and psychotropic drug use at baseline, and (2) analysis of complete cases only, with subsequent correction for the NPI-NH sum score and psychotropic drug use at baseline. Adjustments were made for sex, length of DSCU stay (in months), and time in the study arm (full duration, later enrolment, early drop out, and later enrolment with early drop out; for all cases only). In addition to overall psychotropic drug usage, we performed post hoc analyses for psychotropic drug subgroups: antipsychotics, anxiolytics, antidepressants and hypnotics. We did not perform analyses for anticonvulsants and anti-dementia drugs separately, because of the small sample sizes within these groups. Several models were executed for each subgroup, in line with the analysis of overall usage. The models adjusted for confounders and containing all cases are considered the main models for both the pre-specified and post hoc analyses.

Finally, we conducted sensitivity analyses for the primary and secondary outcomes that considered the results of the process evaluation by excluding nursing homes with tardy or low implementation (*n* = 4) [50].

There were some deviations from the study protocol [45], see Additional file 1.

## Results

### Descriptive data

**Fig. 2:**
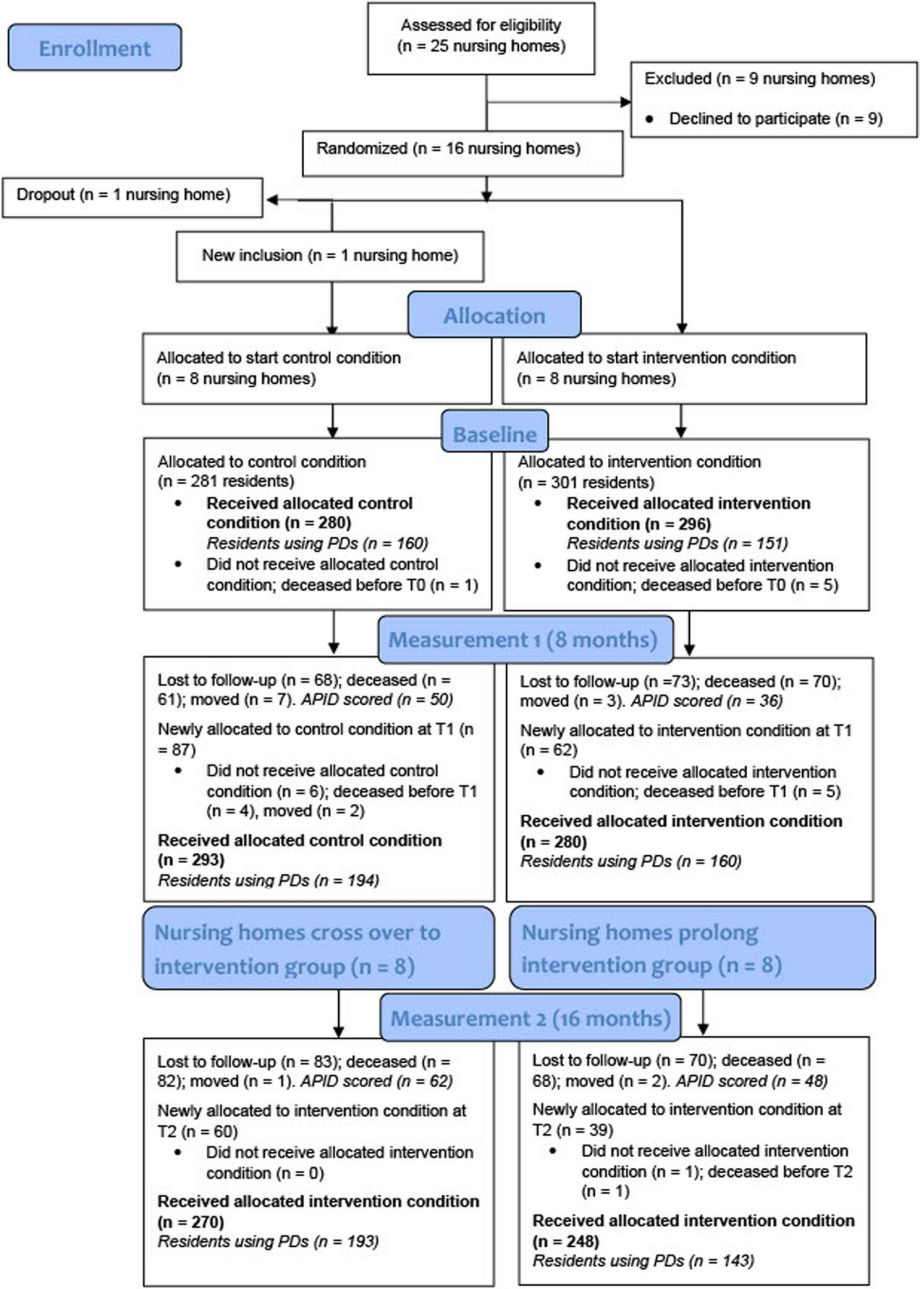
Study flow chart APID = Appropriate Psychotropic Drug Use in Dementia; PD = psychotropic drug

Figure 2 indicates the flow of nursing homes and residents through the study. Of the 25 homes eligible for inclusion between July 2016 and November 2018, nine decided not to participate (before randomization) due to lack of staff commitment or being unable to meet the requirements of participation, leaving sixteen nursing homes available for randomization. One nursing home in the control group also dropped out after randomization, but before the baseline measurements. Therefore, no data were gathered for this nursing home and we recruited a replacement nursing home through our national platform (Vilans Center of Expertise for Long-term Care). There was no loss to follow-up at the cluster level, and loss to follow-up at the resident level did not differ between clusters (control, 46%; intervention, 51%). At baseline, 576 residents participated (control, 280; intervention, 296), of which 311 residents used psychotropic drugs (control, 160; intervention, 151). Thereafter, a total of 236 residents were newly included during the study at the second and third measurement (newly allocated residents minus the dropouts prior to measurement). Hence, in the control group, respectively 81 and 60 residents were newly included at both measurements (total control, 141) and in the intervention group 57 and 38 residents were newly included (total intervention, 95). Characteristics were similar between the control and intervention groups at baseline and for the newly recruited residents at the second and third measurements (see Additional files 2, 3, and 4.

### Primary outcome

An additional file indicates the descriptive data for the mean APID index sum scores at baseline, 8 and 16 months (see Additional file 5). Table 1 regards the effects of the RID intervention on the appropriateness of psychotropic drug use, showing the multilevel model analyses using the APID index sum scores. The crude multilevel model indicated a difference of 0.216 (95% CI: −2.580 to 3.012; *p* = 0.879) on the APID index sum score from baseline to 8 months between the RID intervention and control group. For baseline to 16 months follow-up (i.e., prolonged intervention group and control group crossed over to intervention), this was 1.321 (95% CI: −1.655 to 4.296; *p* = 0.384). The crude effects on the APID index sum score were smaller than the a priori anticipated five points. The results did not change materially after adjusting for confounders or in the sensitivity analyses that excluded the four nursing homes with the lowest implementation levels. Similar numbers of appropriate and inappropriate prescriptions were either stopped or started during the trial.

**Table 1:** Effect of the RID intervention on the appropriateness of psychotropic drug use

	APID index sum score^a^			*P*
	Estimate	95%CI		
		Lower bound	Upper bound	
**Model I: Crude model** Difference between:				
RID intervention and control group from baseline to 8 months	0.216	−2.580	3.012	0.879
RID intervention and re-intervention^d^ from baseline to 16 months	1.321	−1.655	4.296	0.384
**Model II: Including confounders** ^b^ Difference between:				
RID intervention and control group from baseline to 8 months	0.564	−2.449	3.577	0.714
RID intervention and re-intervention^d^ from baseline to 16 months	2.165	−1.113	5.443	0.196
**Model III: Post hoc sensitivity analysis** ^c^ **excl. 4 least performing nursing homes** Difference between:				
RID intervention and control group from baseline to 8 months	0.784	−2.970	4.538	0.682
RID intervention and re-intervention^d^ from baseline to 16 months	2.129	−1.779	6.038	0.286

^a^Theoretical range: 0–102.8. Higher scores indicate less appropriate PD prescribing. The estimates are the differences in APID index sum score between the intervention and control group from baseline to 8 months or from baseline to 16 months. The estimated effect size is approximately 0.33 (in the sample size calculation we aimed for a difference of five points on the APID index sum score between groups and a standard deviation of 15: Van der Spek et al. A reliable and valid index was developed to measure appropriate psychotropic drug use in dementia; Journal of Clinical Epidemiology 2015 [52])

^b^Number of psychotropic drugs per resident, sex, NPI-NH sum score, duration of stay on the unit (in months) and time in the study arm (full duration, later enrolment, early drop out, and later enrolment with early drop out)

^c^Corrected for the abovementioned confounders

^d^control group in phase I, crossed over to intervention in phase II

*APID *Appropriate Psychotropic Drug Use in Dementia, *CI *Confidence interval, *NPI-NH *Neuropsychiatric Inventory-Nursing Home version, *RID *Reducing inappropriate psychotropic drug use

### Secondary outcome

An additional file shows the descriptive data for the percentage of psychotropic drug users, covering overall usage as well as use of psychotropic drug subgroups at baseline, 8 and 16 months (see Additional file 6). No major baseline differences were found between the intervention and control group, although psychotropic drug use was a little higher in the control group. Overall usage was about 50% in the intervention group and about 57% in the control group. Antipsychotics and antidepressants were most frequently used, followed by anxiolytics. The results of the GEE analysis on overall psychotropic drug use are summarized in Table 2. The crude model (Model 1) showed a relatively large intervention effect at 8 months and a larger effect at 16 months. The odds of psychotropic drug usage in the RID intervention group were 0.7 (95% CI: 0.546 to 0.988; *p* = 0.041) and 0.6 (95% CI: 0.460 to 0.839; *p* = 0.002) times as high at 8 and 16 months, respectively. Effect estimates were very similar in the analyses adjusted for confounders (Model 2), though with slightly broader confidence intervals. No large difference in psychotropic drug usage existed between the RID intervention and control group for complete cases (Model 3a and 3b). Again, a greater intervention effect was observed at 16 than at 8 months.

**Table 2:** Effect of the RID intervention on the percentage of psychotropic drug use

	Psychotropic drug use			*P*
	OR	95%CI		
		Lower bound	Upper bound	
**Model 1. Crude model.** Ratio of:				
RID intervention and control group at 8 months	0.734	0.546	0.988	0.041
Both RID intervention groups ^a^ at 16 months	0.621	0.460	0.839	0.002
**Model 2. Including confounders.**^b^ Ratio of:				
RID intervention and control group at 8 months	0.776	0.573	1.051	0.101
Both RID intervention groups ^a^ at 16 months	0.654	0.481	0.889	0.007
**Model 3a. Complete cases only**,** including confounders.**^***c***^ Ratio of:				
RID intervention and control group at 8 months	0.915	0.616	1.358	0.659
Both RID intervention groups ^a^ at 16 months	0.879	0.593	1.305	0.523
**Model 3b. Complete cases only**,** including confounders**^*d*^ Ratio of:				
RID intervention and control group at 8 months	0.836	0.497	1.407	0.500
Both RID intervention groups ^a^ at 16 months	0.745	0.410	1.352	0.333

^a^control group in phase I, crossed over to intervention in phase II

^b^corrected for sex, duration of stay on the unit (in months) and time in the study arm (full duration, later enrolment, early drop out, and later enrolment with early drop out)

^c^corrected for sex, duration of stay on the unit (in months). Not corrected for time in the study arm, since this concerns a subset of data of the complete cases (e.g., full duration). Not corrected for baseline psychotropic drug use and baseline NPI-NH sum score

^d^corrected for sex, duration of stay on the unit (in months), baseline psychotropic drug use, baseline NPI-NH sum score. Not corrected for time in the study arm, since this concerns a subset of data of the complete cases (e.g., full duration)

* Regarding complete cases analyses; there appeared no effect of collinearity between the two variables NPI and PDU

*CI *Confidence interval, *NPI-NH *Neuropsychiatric Inventory-Nursing Home, *OR *Odds ratio, *RID *Reducing inappropriate psychotropic drug use

The sensitivity analysis that excluded four nursing homes produced smaller effect sizes that retained the same directionality (see Models 1 and 2 of Additional file 7). The post hoc analysis on the psychotropic drug subgroups (Table 3) revealed no change on hypnotics and on antipsychotics, although the odds on antipsychotic usage decreased a little. Relatively large effects were found on usage of anxiolytics as well as antidepressants. The odds of anxiolytics usage in the RID intervention group (Model 5, adjusted for confounders) were 0.6 times as high at 8 months (95% CI: 0.430 to 0.952; *p* = 0.027) and about 0.5 times as high at 16 months (95% CI: 0.382 to 0.859; *p* = 0.007) compared to baseline. The odds of antidepressant usage in the RID intervention group (Model 8, adjusted for confounders) were about 0.7 times as high at 8 months (95% CI: 0.478 to 0.973; *p* = 0.035) as well as at 16 months (95% CI: 0.475 to 0.968; *p* = 0.033). Similar effects were found for both subgroups in the crude models. Overall, most of the results showed effect estimates in favor of the intervention, though with variations in effect size and confidence interval width.

**Table 3:** Effect of the RID intervention: post hoc analysis on subgroups (all nursing homes)

Model and ratio	OR	95%CI		*P*
		Lower bound	Upper bound	
**Model 1. Antipsychotics, crude model.**				
RID Intervention and control group at 8 months	0.818	0.581	1.150	0.248
Both RID intervention groups ^a^ at 16 months	0.797	0.565	1.125	0.197
**Model 2. Antipsychotics**,** including confounders.**^**B**^ Ratio				
RID Intervention and control group at 8 months	0.882	0.621	1.254	0.486
Both RID intervention groups ^a^ at 16 months	0.856	0.601	1.219	0.389
**Model 3a. Antipsychotics**,** complete cases only**,** including confounders.**^***C***^ Ratio				
RID Intervention and control group at 8 months	1.444	0.892	2.338	0.135
Both RID intervention groups ^a^ at 16 months	1.500	0.924	2.435	0.101
**Model 3b. Antipsychotics**,** complete cases only**,** including confounders.**^*D*^ Ratio				
RID Intervention and control group at 8 months	1.429	0.765	2.671	0.263
Both RID intervention groups ^a^ at 16 months	1.598	0.809	3.156	0.177
**Model 4. Anxiolytics**,** crude model.**				
RID Intervention and control group at 8 months	0.636	0.428	0.946	0.026
Both RID intervention groups ^a^ at 16 months	0.572	0.382	0.855	0.007
**Model 5. Anxiolytics**,** including confounders.**^**B**^ Ratio				
RID Intervention and control group at 8 months	0.640	0.430	0.952	0.027
Both RID intervention groups ^a^ at 16 months	0.573	0.382	0.859	0.007
**Model 6a. Anxiolytics**,** complete cases only**,** including confounders.**^***C***^ Ratio				
RID Intervention and control groups at 8 months	0.836	0.506	1.381	0.484
Both RID intervention groups ^a^ at 16 months	0.805	0.482	1.342	0.405
**Model 6b. Anxiolytics**,** complete cases only**,** including confounders.**^*D*^ Ratio				
RID Intervention and control groups at 8 months	0.957	0.517	1.774	0.890
Both RID intervention groups ^a^ at 16 months	0.851	0.403	1.797	0.673
		**95%CI**		
**Model and ratio**	***OR***	***Lower bound***	***Upper bound***	***P***
**Model 7. Antidepressants**,** crude model.**				
RID Intervention and control group at 8 months	0.694	0.489	0.984	0.040
Both RID intervention groups ^a^ at 16 months	0.693	0.484	0.992	0.045
**Model 8. Antidepressants**,** including confounders.**^**B**^ Ratio				
RID Intervention and control group at 8 months	0.682	0.478	0.973	0.035
Both RID intervention groups ^a^ at 16 months	0.678	0.475	0.968	0.033
**Model 9a. Antidepressants**,** complete cases only**,** including confounders.**^**C**^ Ratio				
RID Intervention and control group at 8 months	0.703	0.449	1.099	0.122
Both RID intervention groups ^a^ at 16 months	0.796	0.507	1.248	0.320
**Model 9b. Antidepressants**,** complete cases only**,** including confounders.**^***D***^ Ratio				
RID Intervention and control group at 8 months	0.681	0.396	1.173	0.166
Both RID intervention groups ^a^ at 16 months	0.836	0.451	1.551	0.570
**Model 10. Hypnotics**,** crude model.**				
RID Intervention and control group at 8 months	0.976	0.641	1.487	0.911
Both RID intervention groups ^a^ at 16 months	0.855	0.549	1.332	0.488
**Model 11. Hypnotics**,** including confounders.**^**B**^ Ratio				
RID Intervention and control group at 8 months	1.059	0.688	1.630	0.794
Both RID intervention groups ^a^ at 16 months	0.932	0.596	1.457	0.758
**Model 12a. Hypnotics**,** complete cases only**,** including confounders.**^***C***^ Ratio				
RID Intervention and control group at 8 months	1.252	0.692	2.266	0.457
Both RID intervention groups ^a^ at 16 months	0.956	0.508	1.797	0.888
**Model 12b. Hypnotics**,** complete cases only**,** including confounders.**^*D*^ Ratio				
RID Intervention and control group at 8 months	1.625	0.825	3.201	0.160
Both RID intervention groups ^a^ at 16 months	0.951	0.391	2.314	0.912

^a^control group in phase I, crossed over to intervention in phase II

^b^corrected for sex, duration of stay on the unit (in months) and time in the study arm (full duration, later enrolment, early drop out, and later enrolment with early drop out)

^c^corrected for sex, duration of stay on the unit (in months). Not corrected for time in the study arm, since this concerns a subset of data of the complete cases (e.g., full duration). Not corrected for baseline usage of the psychotropic drug subgroup and baseline NPI-NH sum score

^d^corrected for sex, duration of stay on the unit (in months), baseline usage of the psychotropic drug subgroup, baseline NPI-NH sum score. Not corrected for time in the study arm, since this concerns a subset of data of the complete cases (e.g., full duration)

- The two subgroups anti-dementia drugs and anticonvulsants were not included in the GEE analysis given their small number of observations. - Regarding the complete cases analyses; there appeared no effect of collinearity between the two variables NPI and psychotropic drug use. *CI *Confidence interval, *NPI-NH *Neuropsychiatric Inventory-Nursing Home version, *OR *Odds ratio, *RID *Reducing inappropriate psychotropic drug use

## Discussion

### Summary of findings

Tailoring interventions to local contexts using PAR did not reduce inappropriate psychotropic drug use, but it did reduce overall usage, especially in the subgroups anxiolytics and antidepressants. Although one would expect a decrease of inappropriate - and an increase of appropriate prescriptions when targeting appropriateness, similar numbers of appropriate and inappropriate prescriptions were either stopped or started during the trial.

### Comparison to literature

The mean APID sum scores in this study ranged from 23.0 to 27.1 (Additional file 5), which is broadly in line with earlier reports showing a mean APID sum score of 26.6 in a comparable setting and with the same inclusion criteria [17]. Baseline psychotropic drug use was 51% in the intervention group and 57% in the control group, which compares favorably with the previously reported frequencies of 61% and 66% in the Netherlands between 2003–2011 [57, 58]. Despite the lower percentage of psychotropic drug users at baseline in our intervention group, we observed a further decrease at 16 months (51.0%, 50.6%, and 48.3% at baseline, 8-, and 16 months, respectively). A more recent study concluded that psychotropic drug use declined from 62.7 to 40.4% over the period 2003–2018 and no reductions were perceived regarding anxiolytic and antidepressant usage. Compared to this study, our baseline use of psychotropic drugs was about 10% higher and we did observe a reduction of anxiolytic and antidepressant usage [13]. The usage of anxiolytics and antidepressants is common [14, 15], and therefore there is room for improvement to strive for a further reduction of anxiolytics and antidepressants.

### Strengths and limitations

This study used an innovative PAR-RCT design containing several elements that promoted implementation based on knowledge from previous research. Therefore, we could target matters known to be important, such as implementing an intervention tailored to the local context and being able to adjust the implementation over time. Using PAR with two cycles enabled us to examine short- and long-term implementation effects and our study is likely to have had value for nursing homes considering that possible solutions are explored and implemented for problems in local nursing home practice in direct cooperation with relevant stakeholders. The process evaluation [50] suggested that our multicomponent RID intervention was well designed, consistent with a review that argued for a comprehensive approach targeting organizational culture and multidisciplinary collaboration [59].

However, several issues warrant further consideration. First, our recruitment process might have selected nursing homes in which staff already had an interest in psychotropic drug use, meaning potentially above-average standards of usual care. Second, we lacked follow-up data for some residents. Given this is consistent with the naturalistic course of people living in nursing homes, and that no differences were found between study groups, we consider this non-selective dropout. Third, full blinding was not feasible and might have biased the results [60]. Fourth, adjusting for baseline differences was not feasible in the multilevel analyses of appropriateness of psychotropic drug use since each single prescription is the level of observation. Instead, we used change scores. These can be less precise and validity issues such as regression to the mean might occur. Fifth, there might have been an underestimation of the effect size of our intervention. APID index scores were slightly higher in the control group as opposed to the intervention group (Additional file 5), leaving less room for improvement. Also, the determination of the indication and evaluation according to the APID index is difficult for psychotropic drugs that were prescribed prior to nursing home admission (see methods). This might have led to increased sum scores at the next measurement indicating less appropriate prescribing. Nevertheless, this applies to the intervention and control groups, meaning there is no selective bias. Sixth, the RID intervention might have been able to reduce the concomitant use of multiple psychotropic drugs, but this was not an outcome. The APID index cannot capture this change and this was neither captured in the percentage of usage, which was defined as a binary outcome. Seventh, psychotropic drugs used pro re nata were not included in data analysis. Finally, we included fewer residents than anticipated; nevertheless, the power was deemed sufficient because the a priori sample size should have relied on the number of prescriptions and not on the number of residents.

### Implications for research and practice

In retrospect, we still underly the importance of a multidisciplinary approach. Caring for residents with dementia and neuropsychiatric symptoms is a team effort and therefore it is important to include all relevant disciplines. Naturally, (prescribing) physicians play a vital part. Although our study did include physicians in the multidisciplinary project team, their actual involvement and influence in practice differed between nursing homes [50]. Moreover, occasionally participating in a project team is not the same as actively reviewing psychotropic drugs and adjusting them when deemed necessary. This may account for the fact that no effect was found on appropriateness. Assessing readiness for change before implementing a practice change can therefore be considered. This could be incorporated as a covariate in statistical models. Hence, we think it is safe to state that when studies aim for reducing (inappropriateness of) psychotropic drug use, the intervention should be a combination of multidisciplinary psychosocial efforts and directly targeting the prescribing behavior among physicians. Taking into account that the domains indication, evaluation and therapy duration contribute the most to the APID index sum score, the biggest gain in more appropriate prescribing probably lies here for physicians. A possible risk of our PAR intervention was that local practice experienced a great deal of freedom, which potentially contributed to the fact that we did not achieve our main aim. Our process evaluation revealed that despite warnings from our research team, many nursing homes tried to implement a large number of actions which did not target appropriateness of use. Instead teams seemed to have focused on implementing psychosocial alternatives to compensate for a decrease of psychotropic drug use [50]. This may explain the reduction in overall psychotropic drug use without a change in the appropriateness of use. Consequently, many good actions are implemented in daily nursing home practice as a result of our PAR intervention that were beyond our aim. Given that our process evaluation suggested improvements such as better multidisciplinary collaboration, future studies may benefit from including additional outcomes directly associated with the chosen interventions, such as the number of multidisciplinary care team meetings or the time spent with a resident [50]. These could indicate improvements in care that are not captured by the metrics used in the current study. There may be potential for the incorporation of theory of change mapping approaches in implementation research, especially in the early stages of intervention development. Using logic models with hypothetical causal pathways will contribute to informed intervention development. This may provide a more detailed understanding of the effective elements and expected changes in relation to a desired outcome, while also highlighting organizational factors, processes and underlying circumstances influencing its implementation [61] Future studies targeting the management of neuropsychiatric symptoms should include (appropriate) psychotropic drug use and (multicomponent), psychosocial alternatives because neuropsychiatric symptoms can worsen when psychotropic drugs are reduced without compensating for this by using alternatives. Nursing home staff may request the prescription of psychotropic drugs. Expectations that benefits of a psychotropic drug outweigh any side effects may contribute to this. Also, due to staff turnover and understaffing, psychosocial interventions are not always accepted as a proper alternative to psychotropic drugs [36]. These factors are often reported as main barriers to the implementation of interventions targeting psychotropic drug use and/or increasing the use of psychosocial interventions in daily practice [19, 30–34]. In an effort to effectively implement a change, nursing homes should continuously invest in engaging new staff.

The association with higher mortality [62], highlights the importance of more appropriate usage. Thus, the decrease in overall usage as a result of our intervention is still highly relevant considering that usage of psychotropic drugs often comes with adverse effects and are at most modestly effective even when prescriptions are in adherence to guidelines [4–7]. It is promising that it is possible to reduce usage of psychotropic drugs or certain subgroups, such as anxiolytics and antidepressents in our study. This finding of our study should encourage nursing home staff and prescribing physicians to rely less on psychotropic drugs and preferably to stop the prescription of psychotropic drugs where possible. Especially, when we take into consideration that there is certain evidence that discontinuation of antipsychotics for example, can be succesful and has no large effect on neuropsychiatric symptoms, cognitive function and quality of life [63]. Given the effects that we found on the subgroups antidepressants and anxiolytics, it could be interesting to further examine which psychotropic drugs were reduced and to take into account the reasons they were prescribed for. Anxiolytics and antidepressants may be prescribed to treat insomnia and sleep disorders. For example, low doses of trazodone (an antidepressant), is frequently being used as a sleeping pill as there is some evidence of beneficial effects on sleep outcomes without serious adverse events [64]. This may also have accounted for the found effects in our studies. Future studies may do well to take this into account.

## Conclusions

The RID intervention ultimately reflected practical considerations, such as “what will help a resident?” and alternatives to compensate for psychotropic drug use, rather than the quality of prescribing. Indeed, although it did not improve the appropriateness of usage, it reduced overall psychotropic drug use at 8 and 16 months in the subgroups anxiolytics and antidepressants. This should encourage nursing home staff to stop the prescription of psychotropic drugs where possible.

## Supplementary Information

Additional file 1. Protocol deviations.

Additional file 2. Characteristics of nursing home residents included at baseline, both overall and for psychotropic drug users.

Additional file 3. Characteristics of newly recruited nursing home residents at T1–all residents and PD users only.

Additional file 4. Characteristics of newly recruited nursing home residents at T2–all residents and PD users only.

Additional file 5. Mean APID index sum scores over time.

Additional file 6. Psychotropic drug use (overall and subgroups) over time.

Additional file 7. Effects of the RID intervention on the percentage of psychotropic drug use sensitivity analysis.

Additional file 8.

## Data Availability

The datasets used and/or analyzed during the current study are available from the corresponding author on reasonable request.

## Abbreviations

APID: Appropriate Psychotropic Drug Use in Dementia
CI: Confidence Interval
CONSORT: Consolidated Standards of Reporting
DSCUs: Dementia Special Care Units
GEE: Generalized Estimation Equation
NPI-NH: Neuropsychiatric Inventory-Nursing Home
OR: Odds Ratio
PAR: Participatory Action Research
RCT: Randomized Controlled Trial
RID: Reducing Inappropriate psychotropic Drug use
SPSS: Statistical Package for the Social Sciences
