# Supplementary material for: Tailored interventions for inappropriate psychotropic drug use in nursing home residents with dementia: participatory action research in a special case of a stepped-wedge cluster randomized controlled trial
Source: BMC Geriatr. 2025 Aug 2;25:581. doi: 10.1186/s12877-025-06206-y (PMC12318394; doi:10.1186/s12877-025-06206-y)
Supplement: Supplementary file 1 — Additional file 1. Protocol deviations. [file 12877_2025_6206_MOESM1_ESM.docx]

**Additional file 1.** Protocol deviations

| We noticed that correcting for baseline differences was not feasible for the multilevel analyses of the appropriateness of psychotropic drug use because residents can use multiple psychotropic drugs. We did not anticipate this a priori. |
| --- |
| The study protocol also specified multilevel analyses for the secondary outcome (frequency of psychotropic drug use), but we used logistic GEE. This was because the multilevel analysis did not converge and the GEE accounted for clustering of repeated measurements within residents and nursing homes (see also *Methods*). In addition, we decided to do a post hoc analyses based on the results of overall psychotropic drug use for the four largest psychotropic drug subgroups (i.e., antipsychotics, hypnotics, anxiolytics, and antidepressants). This was not explicitly stated in the study protocol. |
| Studies completed before starting the analysis reported few associations (Smeets *et al.*, 2018; Van Der Spek *et al.*, 2018). Therefore, we concluded that it would be better to make a more parsimonious model in which four of the least relevant confounders were excluded. In our opinion, these included:   1. Cognitive abilities: excluded due to insufficient variance. DSCUs are specialized in treating residents with advanced stages of dementia. We only included DSCU residents, and all of these were likely to have had severe levels of cognitive impairment. 2. Distress in nurses due to neuropsychiatric symptoms: Distress is strongly correlated with the severity of neuropsychiatric symptoms (Zwijsen *et al.*, 2014), the latter of which is also included in the analysis. 3. Staff attitudes toward the use of new interventions or treatments, 4. Cooperation between staff members, the working conditions, and characteristics of the DSCU.   Both staff attitudes and cooperation between staff members are team level factors. Since we mainly expected an effect of these variables on the degree of implementation, both variables were replaced by an extensive extent of performance score developed for our process evaluation (Groot Kormelinck *et al.*, 2021). We have included this more comprehensive extent of performance variable in our sensitivity analysis. |
| Ultimately, a fifth confounder was excluded (dementia subtype) because no effective baseline differences were found between groups in our data (table S2). |

DSCUs = dementia care in special care units; GEE = generalized estimating equations.

Groot Kormelinck, C. M. *et al.* (2021) “Process evaluation of a tailored intervention to Reduce Inappropriate psychotropic Drug use in nursing home residents with dementia,” *BMC geriatrics*. BMC Geriatrics, 21(414), pp. 1–14.

Smeets, C. H. W. *et al.* (2018) “Psychotropic drug prescription for nursing home residents with dementia: prevalence and associations with non-resident-related factors,” *Aging and Mental Health*. doi: 10.1080/13607863.2017.1348469.

Van Der Spek, K. *et al.* (2018) “Factors associated with appropriate psychotropic drug prescription in nursing home patients with severe dementia,” *International Psychogeriatrics*, 30(4), pp. 547–556. doi: 10.1017/S1041610217001958.

Zwijsen, S. A. *et al.* (2014) “Nurses in distress? An explorative study into the relation between distress and individual neuropsychiatric symptoms of people with dementia in nursing homes,” *International Journal of Geriatric Psychiatry*, 29(4), pp. 384–391. doi: 10.1002/gps.4014.
