# Supplementary material for: Tailored interventions for inappropriate psychotropic drug use in nursing home residents with dementia: participatory action research in a special case of a stepped-wedge cluster randomized controlled trial
Source: BMC Geriatr. 2025 Aug 2;25:581. doi: 10.1186/s12877-025-06206-y (PMC12318394; doi:10.1186/s12877-025-06206-y)
Supplement: Supplementary file 2 — Additional file 2. Characteristics of nursing home residents included at baseline, both overall and for psychotropic drug users. [file 12877_2025_6206_MOESM2_ESM.docx]

**Additional file 2.** Characteristics of nursing home residents included at baseline, both overall and for psychotropic drug users

|  | **Residents using psychotropic drugs** | | **All residents** | |
| --- | --- | --- | --- | --- |
|  | RID intervention  (n = 151) | Control  (n = 160) | RID intervention  (n = 296) | Control  (n = 280) |
| Mean age (years), [SD] (range) | 83.41 [7.02] (61–97) | 82.24 [8.47] (58–103) | 84.50 [7.12] (58–100) | 82.84 [8.04] (57–103) |
| Sex, female, n (%) | 108 (71.5) | 111 (69.4) | 226 (76.4) | 198 (70.7) |
| Length of stay at dementia special care unit (months), [SD] (range) | 21.72 [16.50] (0-80) | 21.08 [18.04] (0-96) | 26.50 [21.77] (0-120) | 22.34 [19.68] (0-102) |
| PDs per resident, n [SD] (range) | 1.68 [0.95] (1–5) | 1.82 [0.96] (1–5) | N.A. | N.A. |
| Diagnosis of dementia, n (%) |  |  |  |  |
| *Alzheimer’s dementia* | 74 (49.0) | 68 (42.5) | 145 (49.0) | 113 (40.4) |
| *Vascular dementia* | 20 (13.2) | 18 (11.3) | 36 (12.2) | 38 (13.6) |
| *Mixed Alzheimer’s/vascular dementia* | 10 (6.6) | 16 (10.0) | 25 (8.4) | 36 (12.9) |
| *Frontotemporal dementia* | 4 (2.6) | 5 (3.1) | 8 (2.7) | 6 (2.1) |
| *Lewy body dementia and Parkinson’s disease* | 10 (6.6) | 12 (7.5) | 10 (3.4) | 16 (5.7) |
| *Other dementia* | 33 (21.9) | 40 (25.0) | 72 (24.3) | 70 (25.0) |

*Dementia diagnosis had one missing in control group.*

Abbreviations: NA = not applicable; PDs = psychotropic drugs; RID = reducing inappropriate psychotropic drug use; SD = standard deviation.
