# Supplementary material for: Tailored interventions for inappropriate psychotropic drug use in nursing home residents with dementia: participatory action research in a special case of a stepped-wedge cluster randomized controlled trial
Source: BMC Geriatr. 2025 Aug 2;25:581. doi: 10.1186/s12877-025-06206-y (PMC12318394; doi:10.1186/s12877-025-06206-y)
Supplement: Supplementary file 3 — Additional file 3. Characteristics of newly recruited nursing home residents at T1–all residents and PD users only. [file 12877_2025_6206_MOESM3_ESM.docx]

**Additional file 3.** Characteristics of newly recruited nursing home residents at T1–all residents and PD users only

| **Newly included residents at measurement 2** | **Residents with PD** | | **All residents** | |
| --- | --- | --- | --- | --- |
|  | RID Intervention | Control | RID Intervention | Control |
| Number | 29 | 43 | 57 | 81 |
| Mean age (years), [SD] (range) | 82.97 [8.72] (62–96) | 81.40 [7.18] (64–97) | 83.77 [7.37] (62–96) | 81.64 [7.55] (61–97) |
| Sex, female, n (%) | 20 (69.0) | 21 (48.8) | 39 (68.4) | 45 (55.6) |
| Length of stay in DSCU at (months), [SD] (range) | 4.10 [2.41] (0-8) | 6.53 [9.05] (0-43) | 7.09 [12.57] (0-74) | 6.42 [10.02] (0-70) |
| PDs per resident (number), [SD] (range) | 1.34 [0.61] (1-3) | 1.81 [0.93] (1-4) | N.A. | N.A. |
| Dementia diagnosis, n (%) |  |  |  |  |
| *Alzheimer’s dementia* | 9 (31.0) | 17 (39.5) | 20 (35.1) | 35 (43.2) |
| *Vascular dementia* | 3 (10.3) | 5 (11.6) | 4 (7.0) | 7 (8.6) |
| *Mixed Alzheimer’s/vascular dementia* | 4 (13.8) | 7 (16.3) | 10 (17.5) | 15 (18.5) |
| *Frontotemporal dementia* | 2 (6.9) | 0 (0.0) | 2 (3.5) | 1 (1.2) |
| *Lewy body dementia and Parkinson’s disease* | 0 (0.0) | 3 (7.0) | 1 (1.8) | 4 (5.0) |
| *Other dementia* | 11 (38.0) | 11 (25.6) | 20 (35.1) | 19 (23.5) |

DSCU = dementia special care unit; n = number; PD = psychotropic drug; SD = standard deviation.
