# Supplementary material for: Tailored interventions for inappropriate psychotropic drug use in nursing home residents with dementia: participatory action research in a special case of a stepped-wedge cluster randomized controlled trial
Source: BMC Geriatr. 2025 Aug 2;25:581. doi: 10.1186/s12877-025-06206-y (PMC12318394; doi:10.1186/s12877-025-06206-y)
Supplement: Supplementary file 4 — Additional file 4. Characteristics of newly recruited nursing home residents at T2–all residents and PD users only. [file 12877_2025_6206_MOESM4_ESM.docx]

**Additional file 4.** Characteristics of newly recruited nursing home residents at T2–all residents and PD users only

| **Newly included residents at measurement 3** | **Residents with PD** | | **All residents** | |
| --- | --- | --- | --- | --- |
|  | RID Re-intervention | RID intervention | RID Re-intervention | RID intervention |
| Number | 17 | 42 | 38 | 60 |
| Mean age (years), [SD] (range) | 82.65 [7.00] (70–94) | 82.05 [7.09] (66–95) | 83.08 [7.92] (61–94) | 83.08 [7.27] (66–95) |
| Sex, female N (%) | 9 (52.9) | 24 (57.1) | 24 (63.2) | 37 (61.7) |
| Length of stay at dementia special care unit (months), [SD] (range) | 3.76 [2.46] (0-9) | 4.81 [4.32] (0-26) | 4.79 [3.46] (0-19) | 4.95 [4.11] (0-26) |
| PDs per resident (number), [SD] (range) | 1.47 [0.62] (1-3) | 1.79 [1.07] (1-5) | N.A. | N.A. |
| Diagnosis of dementia, N (%) |  |  |  |  |
| *Alzheimer’s dementia* | 12 (70.5) | 18 (42.9) | 20 (52.6) | 22 (36.7) |
| *Vascular dementia* | 0 (0.0) | 4 (9.5) | 5 (13.2) | 5 (8.3) |
| *Mixed Alzheimer’s/vascular dementia* | 2 (11.8) | 5 (11.9) | 4 (10.5) | 9 (15.0) |
| *Frontotemporal dementia* | 0 (0.0) | 2 (4.8) | 0 (0.0) | 2 (3.3) |
| *Lewy body dementia and Parkinson’s disease* | 1 (5.9) | 4 (9.5) | 2 (5.3) | 4 (6.7) |
| *Other dementia* | 2 (11.8) | 9 (21.4) | 7 (18.4) | 18 (30.0) |

PD = psychotropic drug; RID = reducing inappropriate psychotropic drug use; SD = standard deviation.
