# Supplementary material for: Tailored interventions for inappropriate psychotropic drug use in nursing home residents with dementia: participatory action research in a special case of a stepped-wedge cluster randomized controlled trial
Source: BMC Geriatr. 2025 Aug 2;25:581. doi: 10.1186/s12877-025-06206-y (PMC12318394; doi:10.1186/s12877-025-06206-y)
Supplement: Supplementary file 5 — Additional file 5. Mean APID index sum scores over time. [file 12877_2025_6206_MOESM5_ESM.docx]

**Additional file 5.** Mean APID index sum scores over time

| ***Condition***  ***(Phase I)*** | ***Baseline***  ***[548 PDs]*** | ***8 months***  ***[596 PDs]*** | ***Condition***  ***(Phase II)*** | ***16 months***  ***[562 PDs]*** |
| --- | --- | --- | --- | --- |
| *RID intervention* | 26.08 (15.34) [254] | 24.46 (16.19) [257] | *Re-intervention* | 24.74 (15.89) [233] |
| *Control* | 27.07 (17.01) [294] | 25.38 (16.59) [339] | *RID intervention* | 23.03 (15.60) [329] |

Observed mean APID index sum score (SD) [n of PDs]. Theoretical range: 0–102.8. Higher scores indicate less appropriate PD prescribing

APID = Appropriate Psychotropic drug use In Dementia; PDs = psychotropic drugs; RID = reducing inappropriate psychotropic drug use; SD = standard deviation.
