# Supplementary material for: Tailored interventions for inappropriate psychotropic drug use in nursing home residents with dementia: participatory action research in a special case of a stepped-wedge cluster randomized controlled trial
Source: BMC Geriatr. 2025 Aug 2;25:581. doi: 10.1186/s12877-025-06206-y (PMC12318394; doi:10.1186/s12877-025-06206-y)
Supplement: Supplementary file 6 — Additional file 6. Psychotropic drug use (overall and subgroups) over time. [file 12877_2025_6206_MOESM6_ESM.docx]

| **Additional file 6.**Psychotropic drug use (overall and subgroups) over time | | | | | | |
| --- | --- | --- | --- | --- | --- | --- |
| **Psychotropic drugs** | **Condition**  **RID intervention (phase I) +**  **re-intervention (phase II)** | | | **Control (phase I) +**  **RID intervention (phase II)** | | |
|  |  | | |  | | |
|  | Baseline *n=296* | 8 months *n=316* | 16 months *n=296* | Baseline *n=280* | 8 months *n=343* | 16 months *n=332* |
| **Overall usage** | 151 (51.0) | 160 (50.6) | 143 (48.3) | 160 (57.1) | 194 (56.6) | 193 (58.1) |
| **Antipsychotics** | 71 (24.0) | 72 (22.8) | 70 (23.6) | 76 (27.1) | 88 (25.7) | 81 (24.4) |
| **Anxiolytics** | 50 (16.9) | 47 (14.9) | 43 (14.5) | 56 (20.0) | 70 (20.4) | 71 (21.4) |
| **Antidepressants** | 62 (20.9) | 66 (20.9) | 61 (20.6) | 65 (23.2) | 87 (25.4) | 92 (27.7) |
| **Hypnotics** | 40 (13.5) | 45 (14,2) | 38 (12.8) | 51 (18.2) | 48 (14.0) | 49 (14.8) |
| **Anti-dementia drugs** | 20 (6.8) | 18 (5.7) | 15 (5.1) | 28 (10.0) | 24 (7.0) | 20 (6.0) |
| **Anticonvulsants** | 3 (1.0) | 2 (0.6) | 2 (0.7) | 5 (1.8) | 7 (2.0) | 4 (1.2) |
| The number and percentage n (%) of residents with one or more regular psychotropic drug prescriptions (per subgroup). | | | | | | |

RID = reducing inappropriate psychotropic drug use
