# Supplementary material for: Tailored interventions for inappropriate psychotropic drug use in nursing home residents with dementia: participatory action research in a special case of a stepped-wedge cluster randomized controlled trial
Source: BMC Geriatr. 2025 Aug 2;25:581. doi: 10.1186/s12877-025-06206-y (PMC12318394; doi:10.1186/s12877-025-06206-y)
Supplement: Supplementary file 7 — Additional file 7. Effects of the RID intervention on the percentage of psychotropic drug use sensitivity analysis. [file 12877_2025_6206_MOESM7_ESM.docx]

**Additional file 7.** Effects of the RID intervention on the percentage of psychotropic drug use sensitivity analysis

| **Model and ratio** | **Psychotropic drug use** | | | | |
| --- | --- | --- | --- | --- | --- |
|  | ***OR*** | | **95%CI** | | ***P*** |
|  |  |  | ***Lower bound*** | ***Upper bound*** |  |
| **Sensitivity analysis part I: Four nursing homes excluded** |  | |  |  |  |
| **Model 4. Including confounders. ^B^** Ratio |  | |  |  |  |
| RID Intervention and control group at 8 months | 0.820 | | 0.576 | 1.168 | 0.272 |
| Both RID intervention groups ^a^ at 16 months | 0.639 | | 0.447 | 0.915 | 0.014 |
| **Model 5a. Complete cases only, including confounders. *^C^*** Ratio |  |  |  |  |  |
| RID Intervention and control group at 8 months | 0.946 | | 0.594 | 1.506 | 0.815 |
| Both RID intervention groups ^a^ at 16 months | 0.821 | | 0.516 | 1.308 | 0.407 |
| **Model 5b. Complete cases only, including confounders.** *^D^* Ratio |  |  |  |  |  |
| RID Intervention and control group at 8 months | 0.816 | | 0.448 | 1.484 | 0.505 |
| Both RID intervention groups ^a^ at 16 months | 0.596 | | 0.297 | 1.199 | 0.147 |
| ^a^ control group in phase I, crossed over to intervention in phase II.  ^b^ corrected for sex, duration of stay on the unit (in months) and time in the study arm (full duration, later enrolment, early drop out, and later enrolment with early drop out).  ^c^ corrected for sex, duration of stay on the unit (in months). Not corrected for time in the study arm, since this concerns a subset of data of the complete cases (e.g., full duration). Not corrected for baseline psychotropic drug use and baseline NPI-NH sum score.  ^d^ corrected for sex, duration of stay on the unit (in months), baseline psychotropic drug use, baseline NPI-NH sum score. Not corrected for time in the study arm, since this concerns a subset of data of the complete cases (e.g., full duration).  * Regarding the complete cases analyses; there appeared no effect of collinearity between the two variables NPI and psychotropic drug use.  CI = confidence interval; NPI-NH = Neuropsychiatric Inventory-Nursing Home version; OR = odds ratio; RID = reducing inappropriate psychotropic drug use. | | | | | |
